# Competitive floorball over the last decade: a scoping review

**DOI:** 10.3389/fspor.2026.1753340

**Published:** 2026-05-14

**Authors:** Edita Krejzová, Noemi Knapová, Jiří Nykodým, Marta Gimunová

**Affiliations:** 1 Department of Sport Performance and Exercise Testing, Faculty of Sports Studies, Masaryk University, Brno, Czechia; 2 Department of Physical Activities and Health Sciences, Faculty of Sports Studies, Masaryk University, Brno, Czechia

**Keywords:** floorball, review, scoping review, sport science, team sports

## Abstract

**Background:**

Floorball is a rapidly developing sport worldwide, and it is a major contributor to the landscape of competitive indoor sports.

**Objective:**

Despite its rapid growth and international presence, floorball remains underrepresented in sport science literature. This scoping review aimed to summarize peer-reviewed research in competitive floorball from the past decade (2014–2024), categorize studies, and identify gaps to guide future research.

**Methods:**

A systematic search in Web of Science, PubMed, and Scopus identified 310 studies; 55 met the inclusion criteria and were thematically analysed.

**Results:**

Studies were divided into six categories: health related (injury, prevention, overuse, pain, biomarkers, illness, coaches voice), psychology and sociology, physical conditioning and performance, skills, equipment, and nutrition. Most work focused on injury epidemiology and prevention, often emphasizing youth and female athletes. Fewer studies addressed psychology, performance or skills, while nutrition and equipment were each represented by one article. Many relied on cross-sectional or small-sample designs, with limited longitudinal or interventional approaches.

**Conclusions:**

This review maps the fragmented yet developing body of floorball research. While progress has been made in health domains, especially injury prevention, major gaps remain in performance analysis, training methodology, tactical behaviour, recovery, and psychological aspects. Future research should use more diverse and robust designs to strengthen the evidence base for this growing sport.

## Introduction

Floorball is a rapidly developing sport worldwide, particularly in Sweden, Finland, Switzerland and the Czech Republic, where it has established itself as a major contributor to the landscape of competitive indoor sports (1). According to the International Floorball Federation (IFF), there are approximately 330,000 registered players globally (2). Floorball emerged as an organized sport in Gothenburg, Sweden, in 1968. Similar forms of the game had previously been played in the United States, particularly in Minneapolis during the late 1950s and1960s under the name “floor hockey.” The modern game was subsequently formalized in Sweden and rapidly expanded. A key milestone was the founding of the International Floorball Federation (IFF) on April 12, 1986, by Sweden, Finland, and Switzerland (3). As the sport continues to grow, it is gaining recognition in international competitions and expanding beyond its traditional strongholds. The existing body of research on floorball has largely focused on injury epidemiology and health related aspects, with particular emphasis on injury incidence. However, despite floorball's increasing popularity, scientific research on this sport remains limited compared to other well established team sports.

The existing body of research on floorball has largely focused on injury epidemiology and health related aspects, with particular emphasis on injury incidence. Early epidemiological studies on floorball injuries have consistently reported that injuries occur more frequently during matches than during training and predominantly affect the lower extremities, particularly the knee and ankle (4–6). Most injuries have been described as traumatic in nature, with ligament sprains representing one of the most common injury types, although overuse injuries have also been identified (7). Subsequent research has further highlighted a high incidence of injuries in competitive floorball, particularly among female players, and has emphasized the relevance of non-contact injury mechanisms. In addition to lower-limb injuries, studies have also identified eye and orofacial injuries as important health concerns in floorball, suggesting a broader spectrum of injury risk within the sport (8, 9). Research has also addressed injury prevention and risk factors, with early intervention studies demonstrating that neuromuscular training programmes can reduce the risk of non-contact lower-limb injuries while also improving performance-related factors such as balance, agility, and muscle power (10, 11). Furthermore, environmental and equipment-related factors, including playing surface and footwear characteristics, have been identified as potential contributors to injury risk in floorball. In addition to injury related research, studies have also examined broader health aspects in floorball players, including respiratory conditions such as asthma and exercise induced bronchoconstriction, with evidence suggesting lower prevalence compared to athletes participating in cold-environment sports (12).

This evidence suggests that floorball can play an important role in addressing key issues in sports science, including athlete conditioning, injury prevention, and game performance optimization (1). Therefore, this scoping review aimed to map the last decade state of competitive floorball research, categorize existing studies, and identify gaps in knowledge that can guide future research. By synthesizing findings across different areas, this review provided a deeper understanding of the sport and offered a foundation for advancing training methodologies, injury prevention strategies, and performance optimization in floorball.

## Methods

This scoping review was conducted following the framework proposed by Arksey & O'Malley (13), with refinements by Levac et al. (14) and was guided by the PRISMA-ScR (Preferred Reporting Items for Systematic Reviews and Meta-Analyses Extension for Scoping Reviews) checklist (15). The aim of this review was to map the available literature on competitive floorball over the last decade (2014–2024). The review covered all studies that examined competitive floorball outcomes, published between 1^st^ January 2014 and 25^th^ November 2024 in English or Czech. The population of interest comprised competitive floorball players, coaches, and related personnel. The PCC criteria consisted of P/population/: floorball players, and related personnel; C/concept/: different fields of research related to floorball; C/context/: competitive level of floorball.

### Literature search strategy

A comprehensive literature search was conducted by one reviewer (EK) in three electronic databases: Web of Science, PubMed, and EBSCO SportDiscus. The search identified a total of 310 studies, with 134 results from Web of Science, 97 from PubMed, and 79 from EBSCO SportDiscus. The search strategy was based on the following keywords: “floorball” OR “salibandy” OR “innebandy”, ensuring coverage of all relevant terms for the sport. The search was restricted to studies published between 1^st^ January 2014 and 25^th^ November 2024, without applying additional filters for methodology or participant demographics to maximize inclusivity. The exclusion criteria were recreational level, or non-organised floorball (e.g., physical education, playground floorball), non-English or Czech language, review articles, conference papers, books, and book chapters, and no full text available. The search was conducted on 25^th^ November 2024, and all retrieved records were exported to Rayyan (16) for further screening.

**Figure 1:**
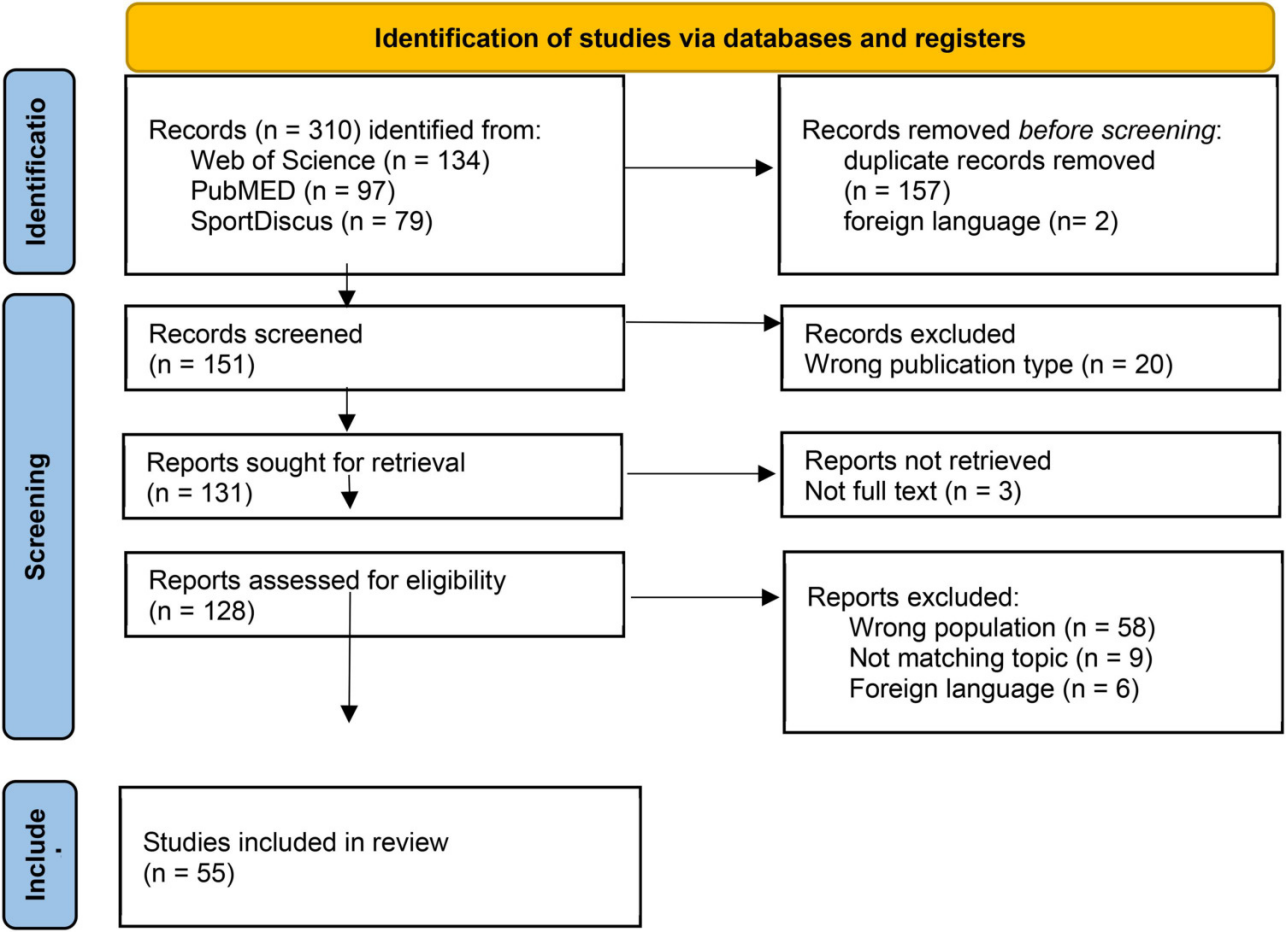
PRISMA flow diagram describing the selection process. Adapted from PRISMA 2020 flow diagram template (80), licensed under CC BY 4.0.

The selection process followed a two-step screening approach. First, duplicate records were removed, and studies were screened based on language, study type, and population. Then, the title and abstract screening was performed to assess the relevance of articles retrieved from the database search. In the second step, full text screening was conducted on the remaining articles to determine their eligibility according to the predefined inclusion and exclusion criteria. Studies were further categorized into thematic sections: health related (injury, injury prevention, overuse injury, pain, biomarkers, illness and voice), physical conditioning and performance, skill, nutrition, psychology and sociology, equipment. The screening process was carried out independently by two reviewers (EK and NK), and any discrepancies were resolved through discussion or consultation with a third reviewer (MG). Studies that did not meet the eligibility criteria were excluded. The selection process was visualized using the PRISMA flow diagram (Figure 1.).

### Data extraction process

Data extraction was conducted by one reviewer (EK) and independently verified by a second reviewer (NK) to ensure accuracy and consistency.

The following key data items were extracted from each included study: study characteristics (author, year, and country), aim and study design, participants (sample size, gender, and age), methodology (measurement tools and follow-up period), and outcomes (key results).

A narrative synthesis approach was used to categorize and summarize key findings from the included studies. The results were grouped thematically based on common research topics identified in the literature, such as health related, physical conditioning and performance, skill, nutrition, psychology and sociology, equipment. Findings were synthesized without statistical meta-analysis due to the heterogeneity of study designs and outcome measures.

## Results

**Figure 2:**
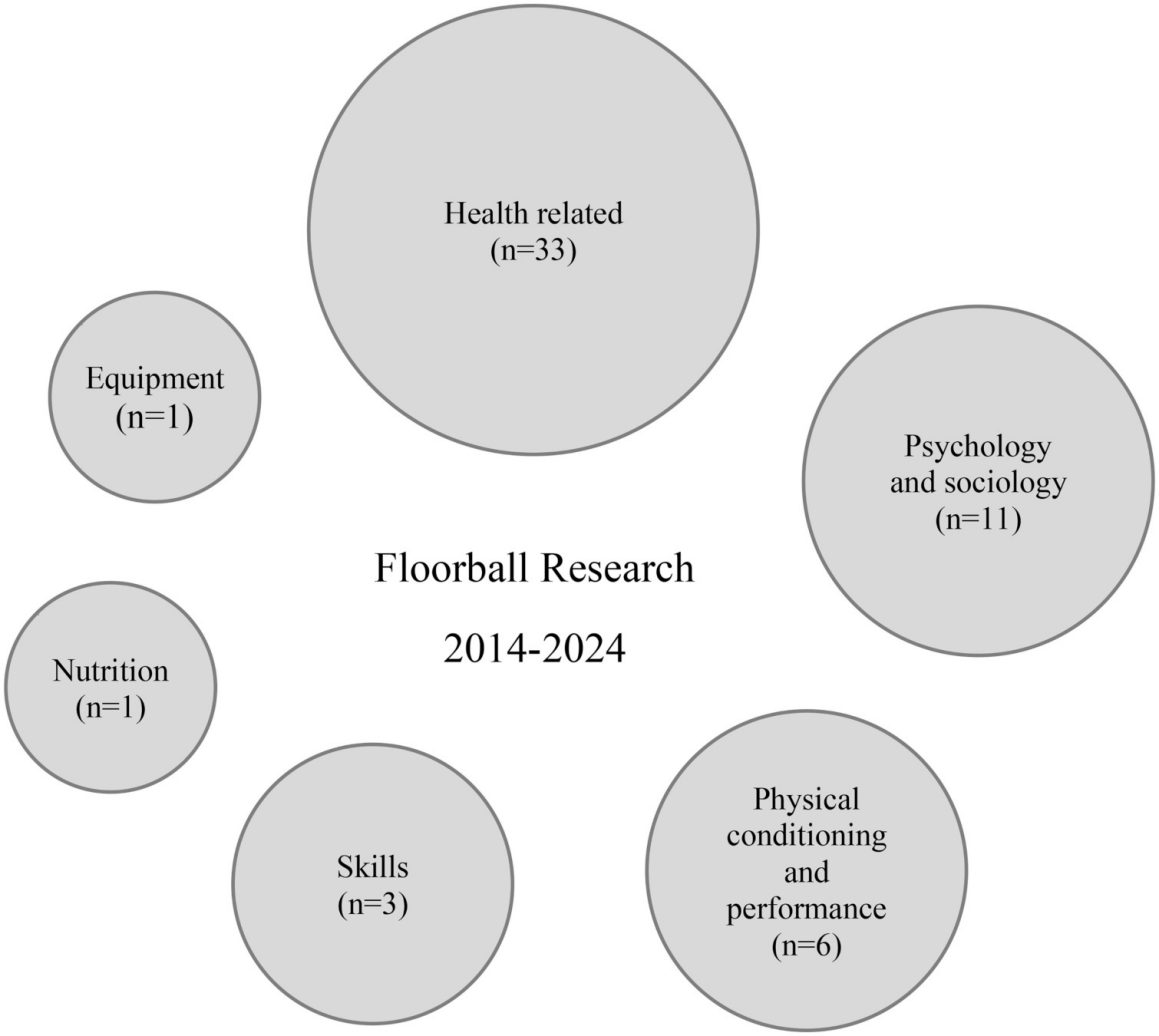
Distribution of research categories in floorball 2014−2024.

This scoping review identified and included a total of 55 studies published between 2014 and 2024, focusing on various aspects of competitive floorball. The included studies varied in design, population characteristics, and research focus, and were grouped into thematic categories to facilitate synthesis. The distribution of studies across categories (Figure 2.), within each category, findings were summarized narratively and supported by key data extracted from the original studies.

Across the 55 included studies, the reported sample size was *N* = 978,880 participants (14,216 participants across the individual study samples. Additionally, the study by Åman et al. (17) analyzed all licensed floorball athletes in Sweden during the years 2006 to 2013. In total, study analyzed 964,664 registrations in floorball; the mean number per year was 120,583 registered athletes (17). Several publications likely used overlapping datasets (e.g., PROFFITS, Knee Control), therefore the summed sample size may include repeated participants across studies. Sample size could not be determined for Åman et al. (18), Rossi et al. (19), Ruoranen et al. (20) studies; these studies were included in the synthesis but excluded from the total sample size calculation.

## Health related studies

The health related category covered injury, injury prevention, overuse injury, pain, biomarkers, illness and voice of coaches as a risk to vocal health (Table 1).

**Table 1:** Health related.

Author	Country	Aim/Purpose of study	Type of study	Participants		Methodology		Results
				Sample size, gender	Age	Methods	Follow-up period	
Injury studies								
Tranaeus et al. (21)	Sweden	Estimate the economic costs of injuries.	Prospective cost analysis	346 players (*M* = 174; *F* = 172)	23.4 ± 4.2 (*M* = 24.4 ± 4.2; *F* = 22.3 ± 3.9)	Injury reporting by team medical staff, cost questionnaire to players and clubs.	1 year	The average cost per injury ranged from 332 to 2,358 Euros, increasing with severity. Overuse injuries were costlier than traumatic injuries for mild cases, but severe traumatic injuries had the highest costs. Knee injuries were the most expensive (1982 Euros per injury). Estimated total cost for Swedish elite floorball injuries was 316,400 Euros per season.
Tranaeus et al. (22)	Sweden	Determine the incidence, severity, and type of injuries.	Prospective cohort study	238 (*M* = 122 male; *F* = 116)	*M* = 25 ± 4.6; *F* = 22 ± 4.2	Injury reporting by team medical staff, exposure tracking.	1 year	Female players had a higher injury incidence (3.9 vs. 2.6 per 1,000 hours, *p* = 0.02). Most common injury sites: thigh (men) and ankle (women). Overuse injuries were more common in men (back issues), while traumatic injuries were more common in women (ankle, knee, ACL). Most injuries were mild. Women had significantly more ACL injuries (11 vs. 2 in men).
Åman et al. (18)	Sweden	Examine incidence and severity of acute injuries using insurance data.	Epidemiological study	–	20.5 ± 7.2 (*M* = 21.3 ± 7.6; *F* = 19.0 ± 6.3)	Insurance claims analysis	4 years	9.2 injuries per 1,000 floorball athletes. Higher injury risk in females (RR 1.4).
Åman et al. (17)	Sweden	Identify the incidence and body location of acute sport injuries in seven sports using a national insurance database.	Cross-sectional study	964664 (*M* = 710 230; *F* = 254 434) floorball players	21 ± 9 (*M* = 21 ± 7; *F* = 20 ± 6) all team sports	Analysis of insurance claims from 2006−2013 in Sweden.	7 years	Floorball had a low overall injury risk but higher risk for head/neck injuries in females. Lower limb injuries were most common in team sports except ice hockey, where head/neck injuries were more frequent. Females generally had a higher risk of lower limb injuries than males. Injury prevention should focus on lower limb injuries in team sports.
Leppänen et al. (23)	Finland	To investigate sagittal plane hip, knee, and ankle biomechanics and ACL injury risk.	Prospective study	75 (*M* = 0; *F* = 75)	16.5 ± 1.8 years	Vertical drop jump test analysed via 3D motion capture. Hip and ankle flexion, knee moments, and ROM were examined.	1–3 years	Landing with less hip flexion ROM and greater knee flexion moment increased ACL injury risk. No association found with ankle flexion or hip flexion moments.
Pasanen et al. (24)	Finland, Switzerland	Investigate the incidence and characteristics of injuries during 12 IFF matches.	Prospective cohort study	2,179 (*M* = 1,100; *F* = 1,079)	24.6 ± 4.8 (*M* = 25.3 ± 4.7; *F* = 23.8 ± 4.7)	Injury data collected by team medical personnel during games and practices.	4 years	Injury incidence in games was 21.24 per 1,000 game hours. Ankle was the most common injury site (24%), followed by head (18%) and knee (18%). Almost half of the injuries (46%) involved joints or ligaments. There was no significant difference in injury incidence between genders.
Pasanen et al. (25)	Finland	To observe incidence and characteristics of acute time-loss injuries.	Prospective cohort study	186 (*M* = 111; *F* = 75)	16.6 ± 1.4	Injury data collected via team diaries and verified by a research physician.	3 years	Injury incidence was significantly higher in games than in practices (26.87 vs 1.25 per 1,000 h). Female players had higher injury rates, especially for joint/ligament injuries.
Tervo et al. (26)	Sweden	Assess whether the 9 + screening test predicts injuries.	Prospective cohort study	84 (*M* = 47; *F* = 37)	21.0 ± 4.2 (*M* = 22.0 ± 4.6; *F* = 19.8 ± 3.1)	9 + screening test, injury tracking	1 season	No association between 9 + screening test score and overall injury risk. Possible link to muscle ruptures/strains but not statistically significant.
Leppänen et al. (27)	Finland	To investigate the association between change of direction biomechanics in a 180degree pivot turn and knee injury risk.	Cohort study	128 (*M* = 79; *F* = 49)	*M* = 6.9 ± 1.4; *F* = 17.5 ± 2.0	Players performed a 180degree pivot turn test while biomechanical variables were measured. Injuries were tracked during follow-up period.	12 months	Female players had a higher rate of knee injuries, including all ACL injuries. Female players showed significantly larger knee valgus angles. None of the investigated variables was associated with knee injury risk.
Castellanos Dolk et al. (28)	Sweden	Examine ACL reconstruction incidence.	Cohort study	7,261 (*M* = 3,256; *F* = 4,005)	*M* = 23.4 ± 4.5; *F* = 21.2 ± 3.9	SNKLR data analysis	10 years	Lower ACL reconstruction incidence in floorball compared to football. Female floorball had lower adjusted ACL—R IR per 1,000 AE (0.85 vs 1.22, IRR = 0.64, 99% CI: 0.48 to 0.86) and adjusted ACL—R IR per 1,000 AS (13.47 vs 19.45, IRR = 0.68, 99% CI: 0.51 to 0.91) compared with football. Male floorball showed a 0.5 times lower IR per 1,000 AE and per 1,000 AS compared with football.
Injury prevention studies								
Åman et al. (29)	Sweden	To examine acute injuries, identifying the most common and severe injuries and recommending injury prevention measures.	Observational study	all licensed players in Sweden	*M* = 21 ± 7; *F* = 19 ± 6	Observational study using national insurance claims data.	10 years	Knee injuries were most common; PMI injuries often involved knee, hand/finger fractures, and eye injuries in floorball. Neuromuscular training and protective eyewear were suggested for prevention.
Perera et al. (30)	Sweden	Describe the motivation for floorball participation, injury prevention, risk perceptions and prevalence of health problems.	Cross-sectional study	471 (*M* = 331; *F* = 140)	*M* = 13.3 ± 1.0; *F* = 13.7 ± 1.5	Baseline survey, OSTRC questionnaire on health problems and overuse injury questionnaire.	1 season	Social aspects were the greatest motivators for participation. Fractures (84% females, 90% males), eye injuries (90% females, 83% males) and concussion (82% females, 83% males) were perceived as the most severe injuries. 93% of players believed that sports injuries can be prevented, while 74% believed it is unlikely that they will sustain an injury.
Åkerlund et al. (31)	Sweden	To evaluate whether the Knee Control injury prevention exercise program reduces injuries.	Cluster randomized controlled trial	471	IG: 13.6 ± 1.1 (*M* = 13.5; *F* = 13.8) CG: 13.2 ± 1.3) (*M* = 13.1; *F* = 13.6)	Weekly injury survey reporting, coach-reported compliance, and physiotherapist visits to verify implementation. Injury data collected using OSTRC.	26 weeks	35% reduction in injury incidence overall and 45% for acute injuries, compared with control group, no preventive effect on overuse injuries
Perera & Hägglund (32)	Sweden	Evaluate exercise and utilisation fidelity of the Knee Control.	Observational study	20 (*M* = 12; *F* = 8)	13.6 ± 1.1	Observation with specific exercise fidelity checklist.	26 weeks	Only 58% of exercises were performed correctly, with higher fidelity in females (71%) than males (54%). Full IPEP completion occurred in only 13% of sessions. Over 90% of coaches believed injuries could be prevented, but only 33% had good knowledge of prevention strategies.
Åkerlund et al. (33)	Sweden	To evaluate player and team compliance with the Knee Control program and its association with injury rates.	Prospective cohort study	301 (*M* = 194; *F* = 107)	13.6 ± 1.1 (*M* = 13.5 ± 1.0; *F* = 13.9 ± 1.3)	Weekly self-reported injury and exposure data, coach-reported compliance, pre/post-season coach surveys.	26 weeks	Higher compliance reduced injury rates: 53% lower overall incidence, 73% lower time-loss injuries in male players. Female players showed no clear injury reduction. High-compliance coaches implemented the full program and progressed exercises more effectively.
Åkerlund et al. (34)	Sweden	To investigate perceptions, facilitators, and barriers regarding the use of the Knee Control injury prevention program.	Cross-sectional survey study	246 players (*M* = 153; *F* = 93); 35 coaches (*M* = 23; *F* = 12)	13.7 ± 1.1 (*M* = 13.5 ± 0.9; *F* = 13.9 ± 1.4)	Pre-season and post-season surveys for players and coaches; analysis based on motivation, self-efficacy, and behavioural intention.	26 weeks	Players saw Knee Control as effective for injury prevention (88%). Facilitators: support, education, player motivation. Barriers: time constraints, lack of space, boring exercises.
Åkerlund et al. (35)	Sweden	To explore perspectives on using the Knee Control.	Qualitative study	42 players (*M* = 18; *F* = 24); 12 coaches	players11–17 years; coaches 39–53	6 focus group discussions with players, 2 with 12 coaches, qualitative analysis.	-	Players found the Knee Control exercises boring but necessary for injury prevention. Coaches struggled with prioritizing injury prevention in time-limited sessions and felt uncertain about their competence.
Overuse injury studies								
Clarsen et al. (36)	Norway	To assess the prevalence and impact of overuse injuries.	Prospective cohort study	50 (*M* = 33; *F* = 17)	sample was 52 floorball players in 2011–2012, 88 in 2012–2013, and 147 in 2013–2014	OSTRC was used weekly during the period.	13 weeks	High prevalence of overuse injuries in floorball, particularly in the knee (27%) and lower back (29%). Most reported injuries had minor impact on performance, but cumulative burden was significant.
Leppänen et al. (37)	Finland	To examine the occurrence, nature, and severity of overuse injuries.	Retrospective study	194 (*M* = 112; *F* = 82)	*M* = 16.9 ± 1.3; *F* = 16.6 ± 2.0	The data were collected using a questionnaire.	3 years	37% players reported at least one overuse injury in the preceding 12 months. Most commonly affecting the lower back/pelvis (39%) and knee (34%). Boys reported significantly more overuse (lower back) injuries than girls (knee).
Leppänen et al. (38)	Finland	Investigate incidence, severity, and risk factors of overuse injuries.	Prospective cohort study	186 (*M* = 111; *F* = 75)	(*M* = 16.9 ± 1.3, *F* = 16.1 ± 1.5)	Injury and exposure tracking, physician interviews	3 years	Floorball players had 114 overuse injuries. The incident of overuse injuries was 1.61 (95% CI 1.32 to 1.91). Higher incidence in females.
Pain studies								
Pasanen et al. (39)	Finland	Investigate the prevalence and risk factors of LBP.	Cross-sectional study	194 (*M* = 112; *F* = 82)	16.8 ± 1.6 (*M* = 16.9 ± 1.3; *F* = 16.6 ± 2.0)	Structured questionnaire on LBP	3 years	LBP has mostly gradual character, shows during floorball training/playing, pain episodes were most prevalent during competitive season, 14% LBP by body contact. LBP prevalence was higher in floorball players (62%) than in basketball players (44%).
Rossi et al. (40)	Finland	Investigate the incidence and risk factors for back pain.	Prospective cohort study	193 (*M* = 111; *F* = 82)	16.8 ± 1.6 (*M* = 16.9 ± 1.3; *F* = 16.5 ± 1.9)	Weekly injury reporting, exposure data from coaches, baseline physical tests	1–3 years	The incidence of non-traumatic back pain was 75 per 1,000 athlete-years in floorball. Nearly half (46%) of non-traumatic cases resulted in over 29 days of missed training. Floorball players had a higher prevalence (62%). No association between LBP and lower extremity strength measures.
Rossi et al. (19)	Finland	Investigate the association between hip-pelvic kinematics, vGRF, LBP.	Prospective cohort study	-	15.7 ± 1.8 (both sports)	SLVDJ, VDJ, motion analysis, injury reporting.	3 years	FPA during SLVDJ increased LBP risk. Right leg FPA <80° was associated with 2.2x higher LBP risk. No significant relationship found between LBP and vGRF.
Rossi et al. (41)	Finland	Investigate the association between pelvic kinematics during the SKL test and LBP.	Prospective cohort study	130 (*M* = 80; *F* = 50)	(*M* = 16.9 ± 1.3; *F* = 17.3 ± 1.8)	SKL test, motion analysis, weekly injury reporting.	1 year	SKL test pelvic movement was not associated with future LBP. LBP incidence was 12% in floorball players. Most cases had a gradual onset and led to moderate time loss from training (median 14 days).
Buková et al. (42)	Slovakia	Assess prevalence and severity of upper extremity pain and disability.	Cross-sectional study	*M* = 61	25.31 ± 3.83	Standardized pain and disability questionnaires (SPI, OES, PRWE).	15 months	Floorball players had the highest elbow pain prevalence, lowest shoulder pain, and highest wrist pain rates.
Hagovská et al. (43)	Slovakia	Compare prevalence and risk of LBP in professional football, ice hockey, and floorball athletes.	Cross-sectional study	*M* = 61	25.31 ± 3.83	ODI questionnaire	15 months	No significant differences in ODI scores between groups.
Hagovská et al. (44)	Slovakia	Determine prevalence of lower limb pain and disability.	Cross-sectional study	*M* = 61	25.31 ± 3.83	Standardized questionnaires (OHS, IKDC, FADI).	Single session	Floorball players had the highest knee (81.9%) and ankle (62.3%) pain prevalence.
Biomarkers studies								
Wedin & Henriksson (45)	Sweden	Investigate postgame elevation and reproducibility of cardiac biomarkers.	Experimental study	*M* = 23	16–34	Blood samples taken pregame, immediately postgame, and 2 h postgame for CK-MB, myoglobin, and hs-cTnT analysis.	3 months	Significant postgame elevations in all cardiac markers. hs-cTnT exceeded myocardial damage cutoff (≥14 ng/L) in the same 6 players after both games. Findings suggest exercise-induced cardiac marker elevations are common but physiological, requiring further investigation.
Wedin & Henriksson (46)	Sweden	Examine the effect of floorball on hematological parameters relevant for health assessment and antidoping testing.	Observational study	*M* = 23	18–23	Blood samples collected pregame, immediately postgame, and 2 h postgame.	Single session	Haemoglobin decreased from 146 pregame to 141 g/L postgame, recovering within 2 h. WBC count increased from 7.2 to 10.1 × 10⁹/L. OFF-hr score decreased, impacting antidoping test interpretation. Exercise-induced changes in hematological markers should be considered in antidoping protocols.
Martusevich et al. (47)	Russia	Study the peculiarities of HRV and microcirculation.	Cross-sectional study	47	18–21	ECG registration, hemodynamic assessment using MS FIT Pro.	Single session	Track and field athletes showed better myocardial fitness and larger cardiovascular adaptive resources than floorball players. Floorball players had relative tachycardia and higher systolic blood pressure. Floorball players exhibited increased sympathetic stimulation of the myocardium, suggesting a higher risk of arrhythmogenic.
Illness studies								
Perera et al. (48)	Sweden	To describe weekly illness prevalence and illness symptoms.	Prospective cohort study	471 (*M* = 329; *F* = 142)	*M* = 13.3 ± 1.0; *F* = 13.7 ± 1.5	Weekly online survey OSTRC questionnaire on health problems.	26 weeks	61% of youth floorball players reported at least one illness week during the season. The prevalence was slightly higher among females than males. In total, 49% (53% male, 43% female) of illness reports indicated that the player could not participate in floorball. Fever (30%), sore throat (16%) and cough (14%), nasal symptoms (13%) were the most common.
Sonesson et al. (49)	Sweden	Investigate risk factors for injury and illness.	Prospective cohort study	471 (*M* = 329; *F* = 142)	*M* = 13.3 ± 1.0; *F* = 13.7 ± 1.5	Weekly surveys on stress, sleep, well-being, training load, injury, and illness occurrence.	26 weeks	Higher stress, poorer sleep, and lower well-being increased injury risk by 8–10% per unit increase. Stress and poor well-being also increased illness risk (8–12%).
Vocal studies								
Buckley et al. (50)	Australia	collaboratively understand coaches’ vocally reliant occupational participation and consider vocal ergonomic factors.	exploratory study	3	34,33	fieldnotes, interviews, observations, a workshop, ergonomics approaches, and a focus group. Multi-level analyses were used.	7 days	Coaches identified personal, environmental, activity-based, and organizational vocal risk factors. Four vocal ergonomics strategies were co-developed and trialed: (1) player consultation, (2) ongoing feedback, (3) increased movement/postural change, and (4) task adaptations. Coaches reported improved awareness and actionable insights for supporting vocal health.

ACL, anterior cruciate ligament; ACL-R, anterior cruciate ligament reconstruction; AE, athlete exposures; AS, athlete seasons; CI, Confidence Interval; CK-MB, Creatine kinase MB; ECG, Electrocardiogram; FADI, Foot and Ankle Disability Index; FPA, femur-pelvic angle; HRV, heart rate variability; hs-cTnT, high-sensitivity cardiac troponin T; IKDC, International Knee Documentation Committee; IPEP, injury prevention exercise programme; IR, incidence, rate; LBP, low back pain; MB, myoglobin; MS FIT Pro, “Medical Soft” sports testing system; ODI, Oswestry Disability Index; OES, Oxford Elbow Score; OHS, Oxford Hip Score; OFF-hr, OFF-score for haemoglobin and reticulocytes; OSTRC, Oslo Sports Trauma Research Centre; OSTRC, Overuse Injury Questionnaire; OSTRC, Oslo Sports Trauma Research Centre; PRWE, Patient Rated Wrist Evaluation; ROM, range of motion; RR, risk ratio; SKL, standing knee lift.; SLVDJ, single-leg vertical drop jump; SNKLR, Swedish National Knee Ligament Registry; SPI, Shoulder Pain and Disability Index; VDJ, vertical drop jump; vGRF, vertical ground reaction force; WBC, white blood cells;.

### Injury

Injury incidence and characteristics were reported in ten of the included studies, mostly conducted in Sweden and Finland. Study designs included prospective cohort studies, cost analyses, and epidemiological studies, with sample sizes ranging from 75 to over 7,000 participants. The age of participants varied, covering youth and adult players.

Overall injury incidence ranged from 1.25 to 26.9 injuries per 1,000 hours. Higher rates were consistently reported during games compared to trainings. For example, Pasanen et al. (25) found a higher injury rate in junior teams matches than in practices (26.87 vs. 1.25 per 1,000 hours). Pasanen et al. (24) found a rate of 21.24 per 1,000 game hours during international floorball matches.

Several studies highlighted a higher injury risk in female players, particularly regarding knee and ACL injuries (22, 26–28, 51). Female athletes demonstrated greater knee valgus angles and biomechanical differences during landing and cutting movements (27, 51). Castellanos Dolk et al. (28) showed that ACL reconstruction incidence was lower in floorball than in soccer, but female floorball players still had a substantially higher rate of ACL injury compared to male players.

The most commonly injured areas were the ankle, knee, thigh and head/neck (17, 25). Overuse injuries were reported more frequently in men (especially back), while traumatic injuries were more prevalent in women (22). Nearly half of all reported injuries involved joints or ligaments (24). While many injuries were mild, their cumulative burden was significant. Reduced hip flexion and increased knee flexion moments during landings were associated with elevated ACL injury risk (27, 51). Screening tools such as the 9+ test score reflected strength, stability, mobility, and functional movement pattern with an emphasis on the lower body and core. The test did not demonstrate predictive value for injury risk in elite floorball players (26).

Additionally, Traneaus et al. (21) conducted a cost analysis estimating that the average cost per injury ranged between €332 and €2,358, The mild and moderate overuse injuries were costlier than the corresponding traumatic injuries. However, the severe traumatic injuries were associated with higher costs than overuse injuries, with knee injuries being the most expensive. The total annual injury-related cost, for 1 year, 346 players in Swedish elite floorball was estimated at €316,400.

### Injury prevention

Injury prevention strategies in floorball were explored in seven studies conducted in Sweden, using a variety of designs including cohort studies, randomized trials, and qualitative or observational approaches. Sample sizes ranged from 20 to 471 participants and one study reported all licenced players in 10 years. The age of participants varied, covering youth and adult players, and adult coaches.

The effectiveness of the Knee Control program (31), found a 35% reduction in injury incidence overall and a 45% reduction in acute injuries in the intervention group. However, there was no preventive effect on overuse injuries. Similarly, Åkerlund et al. (33) that high compliance with the program led to a 53% reduction in overall injury rate and a 73% reduction in time-loss injuries among male players. Notably, no clear injury reduction was observed in female players.

Adherence to the program was low, as only 58% of exercises were performed correctly, and full program completion occurred in just 13% of sessions (32). While fidelity was higher among females (71%) than males (54%), although the number of female participants was lower than that of males, implementation quality remained suboptimal. Players and coaches reported that the program was effective, but barriers such as time constraints, lack of space, and repetitive content were commonly mentioned (34, 35). Coaches reported uncertainty regarding their competence and struggled to prioritize injury prevention during limited training time. It was identified social factors as key motivators for participation and found that 93% of players believed injuries can be prevented, though only 33% of coaches had sufficient knowledge about prevention strategies (30). Åman et al. (29) emphasized the need for injury prevention measures targeting knee and eye injuries, recommending neuromuscular training and protective eyewear.

### Overuse injuries

Three studies examined overuse injuries in floorball players, conducted in Norway and Finland, using a variety of designs including cohort studies and retrospective study. The number of participants ranged between 50 and 194.

Clarsen et al. (36) found by the Overuse Injury Questionnaire a high prevalence of overuse injuries, particularly in the knee (27%) and lower back (29%). While most reported injuries had a minor impact on performance, the cumulative burden was significant. In a study by Leppänen et al. (37), 37% of players reported via questionnaire at least one overuse injury in the preceding 12 months. The most affected areas were the lower back/pelvis (39%) and the knee (34%). Boys reported more lower back overuse injuries, while girls reported more knee-related overuse injuries. Leppänen et al. (38) tracked players over three years using exposure data and physician interviews. The incidence rate was 1.61 per 1,000 h of exposure, with a higher incidence observed in females (1.94) than males (1.23). The most affected areas were knee and lower back, mostly muscle/tendon.

### Pain

A total of seven studies, conducted in Slovakia and Finland examined the prevalence, risk factors, and biomechanical associations of pain among floorball players. With a particular focus on upper extremities, lower limbs, and low back pain (LBP). Thee cross-sectional and prospective cohort studies included 61 to 287 participants, youth or adult players.

Upper extremity pain and disability was investigated by Buková et al. (42), the total score were shoulder (1.22 ± 2.60) by Shoulder Pain and Disability Index, elbow (4.21 ± 11.93) by Oxford Elbow Score and wrist (1.98 ± 5.61) by Patient Rated Wrist Evaluation. Lower limb pain and disability was assessed by Hagovská et al. (44), the total score results were hip (56.15 ± 5.92) by Oxford Hip Score, knee (54.49 ± 37.04) by International Knee Documentation Committee and ankle (86.97 ± 34.37) by Foot and Ankle Disability Index.

Low back pain (LBP) was a prominent topic, addressed in five studies (19, 39–41, 43). Hagovská et al. (43) compared LBP across male team sports (football, ice hockey, floorball) and found no significant differences in Oswestry disability index scores (floorball 3.48 ± 4.91).

Pasanen et al. (39) reported that LBP had mainly occurred gradually than suddenly, usually during floorball specific training or playing, less in strength training or others, the pain episodes were most prevalent during competition, 14% of floorball players reported LBP caused by body contact with another player. Rossi et al. (40), reported non-traumatic back pain incidence of 75 per 1,000 athlete-years (0.3 per 1,000 hours of AE). Nearly half of these cases (46%) caused more than 29 days of missed training. Both studies used standardized Nordic questionnaire of musculoskeletal symptoms about LBP and reported higher LBP prevalence in male and female floorball players than in basketball players.

Biomechanical risk factors were further analysed by Rossi et al. (19), showed that a foot progression angle (FPA) <80° during single-leg drop jump was associated with a 2.2× higher LBP risk during right leg landing but not significant in left. No significant link, in first and second study years, was found with vertical ground reaction forces (vGRF), but in the third year of follow-up there was mean peak vGRF higher in players who developed LBP during the follow-up. Fifty-four percent of floorball players had LBP during the follow-up. Rossi et al. (41) observed no association between pelvic motion during the standing knee lift (SKL) test and future LBP, although a 12% LBP incidence was reported, mostly gradual in onset and resulting in moderate time loss (median: 14 days).

### Biomarkers

Three studies investigated cardiovascular and haematological biomarkers in floorball players, focusing on autonomic regulation, cardiac stress markers, and blood parameters relevant for health assessment and antidoping protocols. Two studies were conducted in Sweden and one in Russia, age of participants ranged between 16 and 34 years.

Martusevich et al. (47) conducted a cross-sectional comparison between track and field athletes and floorball players. Floorball players exhibited higher resting heart rate, higher systolic blood pressure, and greater sympathetic nervous system stimulation, which indicated lower myocardial fitness and a potentially higher risk of arrhythmogenic response under physical stress.

Wedin & Henriksson (45) conducted a controlled laboratory study and found significant postgame increases in all measured cardiac biomarkers, including high-sensitivity cardiac troponin T (hs-cTnT). In 6 of the 23 players, hs-cTnT levels exceeded the threshold for myocardial damage (≥14 ng/L) after both observed matches. Despite these elevations, the authors concluded the changes were likely physiological adaptations, but further investigation is warranted. In follow-up study (46), the same authors examined haematological responses. It was founded that haemoglobin dropped from 146 to 141 g/L postgame but normalized within 2 h. White blood cell counts rose significantly (7.2 to 10.1 × 10⁹/L), and the OFF-hr score decreased, potentially complicating the interpretation of antidoping tests. The authors highlighted the importance of considering exercise-induced haematological shifts in anti-doping evaluations.

### Illness

Illness in floorball players was examined in two studies. Both studies were conducted in Sweden and included 329 players.

The two prospective cohort studies involved youth players over a 26-week competitive season. Perera et al. (48) found that 61% of players experienced at least one week of illness during the season. The prevalence was slightly higher in females than males. Notably, 49% of all illness reports led to missed participation in floorball. The most reported symptoms were fever (30%), sore throat (16%), cough (14%), nasal symptoms (13%). Sonesson et al. (49) explored contributing factors and identified that higher stress, poorer sleep quality, and reduced well-being were significantly associated with both increased injury and illness risk. Specifically, for each unit increase in stress or decrease in well-being, the risk of illness rose by 8%–12%, suggesting that psychosocial load played a meaningful role in athlete health.

### Vocal ergonomics

Coaches' vocal ergonomics was analysed in one Australian exploratory study. Factors affecting voice use in coaching included coaching activities, environmental conditions, sport-specific limitations, player factors and personal behaviour. The adverse voice symptoms were hoarseness, and croakiness. Implemented ergonomic strategies included coaches' consultation, ongoing feedback discussions, movement and posture changes, task adaptation. The study highlighted the need for extended implementation, particularly for early career coaches (50).

## Psychology, sociology

A total of 11 studies (Table 2) addressed psychological and sociological aspects of floorball. These studies were conducted predominantly eight in Sweden, with others one from Finland, one from Poland, one from Switzerland, and one from the Netherlands. Study designs were six of qualitative, three followed by intervention, one of observational, and one of longitudinal designs. Participant samples ranged from small groups of eight individuals to larger cohorts exceeding 400 participants, including both youth and adult players, coaches, referees, and federation staff.

**Table 2:** Psychological and sociological studies.

Author (year)	Country	Aim/Purpose of study	Type of study	Participants		Methodology		Results
				Sample size, gender	Age	Methods	Follow-up period	
Stenling & Tafvelin (52)	Sweden	Examine the relationship between transformational leadership and athlete well-being, and whether need satisfaction mediates this relationship.	Qualitative study	184 (*M* = 74; *F* = 110)	17.7 ± 1.76	Self-reported questionnaires on perceived transformational leadership, need satisfaction, and well-being.	Single session	Transformational leadership positively influenced athlete well-being. This effect was fully mediated by need satisfaction. Coaches who provided autonomy, competence support, and relatedness improved athlete well-being.
Stenling et al. (53)	Sweden	Investigate the psychometric properties of sport-specific measures of coaches’ need-supportive and controlling interpersonal styles.	Qualitative study	277 (*M* = 135; *F* = 142)	16.8 ± 1.1	Surveys measuring athletes’ perceptions of coach behaviour, bifactor ESEM analysis.	Single session	The need ISS-C was best represented as a unidimensional measure, with a global factor explaining most of the variance. The CCBS had both a general factor and specific factors, but subscales varied in how variance was distributed.
Tranaeus et al. (54)	Sweden	Evaluation of psychological skills training intervention at the group level aiming to prevent injuries (traumatic and overuse).	Intervention study	401 (*M* = 203; *F* = 198)	*M* = 23.6 ± 4.4; *F* = 21.3 ± 3.8	The intervention group received six psychological training sessions focusing on stress management, goal setting, relaxation, self-confidence, and emotional control.	1 season	In total, 35% of players sustained 197 injuries (0.49 injuries per player). The intervention group had a lower injury rate (0.45 injuries per player) compared to the control group (0.53 injuries per player). However, no significant differences were found between groups. Female players in the intervention group had a lower injury incidence than those in the control group.
Tranaeus et al. (55)	Sweden	Evaluate the effect of a psychological group-based injury prevention program.	Prospective cohort study	346 (*M* = 174; *F* = 172)	22.4 ± 4.2	Injury reporting by medical staff, psychological skills training intervention.	2 seasons	Intervention group had a lower injury rate (0.31 injuries/player) vs. control (0.41 injuries/player). Fewer severe injuries in the intervention group (46% reduction). No significant statistical difference between groups, but a small effect size (Cohen's d = 0.2) in reducing traumatic and overuse injuries.
van der Does et al. (56)	Netherlands	Investigate the effect of physical and psychosocial stress and recovery on field-test performance.	Prospective cohort study	*F* = 10	24.8 ± 4.5	Monitoring of training logs, stress/recovery questionnaires, and field-tests (HIMS & RMAT).	6 months	Increased psychosocial stress and reduced recovery 3−6 weeks before testing led to decreased aerobic endurance HIMS. More physical stress 6 weeks before testing improved agility RMAT. Both physical and psychosocial stress-recovery balance influenced performance.
Larneby (57)	Sweden	Explore and discuss the construction and display of gender in a mixed-sex floorball group.	Qualitative study	21 (*M* = 14; *F* = 7)	12–16 years	Observations 10 lessons and semi-structured interviews (7 players).	2 months	The mixed-sex setting reinforced a “boys are better than girls” discourse. Gender hierarchies were not fully transcended but were somewhat negotiated. Boys often dominated play and girls adapted by striving to play “like boys.” Shared ambitions helped reduce some gender stereotypes.
Elliason (58)	Sweden	Explore children's rights awareness and experiences.	Qualitative study	8 (*M* = 4; *F* = 4) players, 4 male coaches	12–16 years (players), adults (coaches)	Semi-structured interviews with players and coaches.	No follow-up	Limited awareness of children's rights in sport. Coaches and players saw rights as important but not systematically implemented. Adults had more influence over decisions than children.
Gråstén et al. (59)	Finland	Examine the associations and development of motivational climate, achievement goals, and physical functional skills.	Longitudinal study	*M* = 283	11.49 ± 0.27	Physical functional skill tests and self-report questionnaires.	12 months	Task-involving climate linked to mastery approach, ego-involving climate to performance approach. Weaker endurance linked to mastery approach. Performance and physical ability improved over time.
Wagnsson et al. (60)	Sweden	Evaluate the impact of a multi-level intervention program on reducing dropout rates.	Intervention study	*F* = 85 floorball players, 15 coaches, 80 parents	13–18 (players)	Multi-level intervention: club policy changes, coach/parent/athlete education, hiring a youth sport supervisor, forming a youth committee.	2 years	Only 11% of female athletes dropped out, compared to 27% of male players in the same club. Typical annual dropout rates 30–35%. The intervention contributed to improved retention, likely due to policy changes, stakeholder education, and creating a more inclusive and supportive environment.
Firek et al. (61)	Poland	Evaluate referee-player interactions in youth floorball regarding positive climate and responsiveness.	Observational study	21 referees (*M* = 20; *F* = 1)	24.4 ± 4.1	Observation, video recordings, structured analysis.	1 season	Referees scored low in building a positive climate (2.81/7) and medium in responsiveness (3.81/7).
Ruoranen et al. (20)	Switzerland	Examine the negative effects of proactive professionalisation strategies in a national sport federation.	Qualitative study	Swiss Floorball Federation	adults	Semi-structured interviews, document analysis, secondary studies.	3 years	Identified negative consequences of professionalisation: 1) Tensions between volunteers and paid staff, 2) Overwhelmed clubs, 3) Diverging visions between federation and clubs, 4) Weakened legitimacy due to top-down strategies and limited grassroots involvement.

ESEM, exploratory structural equation modeling; ISS-C, need-supportive style; CCBS, controlling interpersonal style; HIMS, Heart rate Interval Monitoring System; RMAT, Repeated Modified Agility T-test.

### Children's rights and interpersonal climate

Eliasson (58) found limited awareness of children's rights in sport among Swedish youth and coaches. Adults held more decision-making power, and while rights were valued, they were not systematically applied. Referees in Poland scored low in creating a positive emotional climate and only moderate in responsiveness (61).

### Motivational climate and gender

Gråstén et al. (59) demonstrated that a task involving motivational climate was associated with mastery oriented goals and improved physical performance over time, while an ego involving climate aligned with performance orientation. Gender dynamics in mixed-gender floorball settings and found reinforcement of traditional gender hierarchies. Boys dominated play, while girls adapted by mimicking male behaviour, although shared goals helped mitigate stereotypes (57).

### Organizational and system-level factors

Unintended negative consequences of proactive professionalisation in the Swiss Floorball Federation. These included internal tensions between volunteers and staff, overwhelmed clubs, and weakened grassroots legitimacy due to top-down governance strategies (20).

### Leadership and coaching climate

Transformational leadership positively influenced athlete well-being, with need satisfaction (autonomy, competence, relatedness) mediating the relationship (52). In a related psychometric study, Stenling et al. (56) demonstrated that the Interpersonal Supportiveness Scale–Coach (ISS-C) was best represented as a unidimensional measure, with a general factor explaining most of the variance in athletes' responses, suggesting limited distinction between the subdimensions of autonomy support, structure, and involvement. In contrast, the Controlling Coach Behaviors Scale (CCBS) exhibited both a general controlling factor and several specific factors, although the amount of variance explained by each varied. Specifically, “controlling use of rewards” appeared as a distinct factor with weak association to the general controlling style, while “intimidation” loaded strongly on the general factor. These results support the dimensional complexity of controlling coaching behaviours but suggest a simpler underlying structure for perceived need support.

### Psychological skills and injury prevention

The first study focused on effect of a psychological skills training program on injury incidence, showed a slightly lower injury rate (0.45 injuries/player) than the control group (0.53 injuries/player), no statistically significant differences were found. The effect size was small (Cohen's d = 0.2) for overuse injuries, and even smaller for traumatic injuries (55).

The second study evaluated the long-term effect of the same intervention. Although no psychological sessions were delivered in the second season, the intervention group continued to show a lower injury incidence (0.31 vs. 0.41 injuries/player in the control group) during the follow-up. Notably, the intervention group experienced 46% fewer severe injuries in the second season. However, none of the between-group differences were statistically significant. The reported effect sizes remained small for both traumatic and overuse injuries (54).

### Stress and performance

The effect of physical and psychosocial stress on performance were high stress and poor recovery 3–6 weeks before testing were linked to lower 6 Heart rate Interval Monitoring System (HIMS) performance, while higher physical stress improved 4 Repeated Modified Agility T-test (RMAT) performance (56).

### Dropout prevention

Wagnsson et al. (60) evaluated a multi-level intervention including policy changes, education, and stakeholder involvement. During the intervention, only 11% of female players dropped out, compared to 27% of their male counterparts in the same club and typical dropout rates of 30%–35% in Swedish youth sport. Although the study lacked a control group, the authors suggest that the low female dropout rate was linked to the intervention.

## Physical conditioning and performance

Six studies (Table 3) focused on physical conditioning and performance in floorball players. These studies were conducted two in the Czech Republic, one in Singapore, one in Singapore and USA, one in Finland, and one in Sweden. Study designs included two randomized controlled trials, one quasi-experimental intervention, two cross-sectional studies, and one observational study. Sample sizes ranged from 9 to 332 participants, with four studies involving male only samples and two including all genders. Participant ages ranged from 14 to 25 years.

**Table 3:** Physical conditioning and performance studies.

Author (year)	Country	Aim/Purpose of study	Type of study	Participants		Methodology		Results
				Sample size, gender	Age	Methods	Follow-up period	
Levinska et al. (62)	Czech Republic	Evaluate the effect of neuromuscular coordination and strength training on stance stability.	Interventional (quasi-experimental)	*M* = 16	*M* = 24.0 ± 3.6	Pre/post measurements using force plate (stabilometery); 5 postural positions with and without visual input.	4 months	The senso-motor coordination and muscle strength training program significantly improved standing stability parameters especially in more demanding postural positions.
Maly et al. (63)	Czech Republic	Investigate morphological and isokinetic strength asymmetry.	Cross-sectional study	*M* = 22	*M* = 14.4 ± 0.5	Anthropometric measurements, bioelectrical impedance analysis, isokinetic dynamometer testing.	single session	Floorball players had the lowest bilateral lower limb asymmetry (0.32 kg) compared to soccer players (0.58 kg) and non-active boys (0.63 kg) but also lower strength of knee extensors.
Wedin, Nyberg & Henriksson (46)	Sweden	Investigate the impact of training specificity on exercise-induced cardiac troponin elevation.	Observational study	*M* = 9	23–25	Two exercise tests: continuous cycle ergometer test and Yo-Yo Intermittent Recovery 2 (Yo-Yo IR2) test; hs-cTnT blood samples at baseline, 0,2,6 and 24 h post-exercise.	Single session	No hs-cTnT elevation above myocardial damage cutoff after the cycle ergometer test, but 3 out of 9 players exceeded the cutoff after the Yo-Yo IR2 test. Peak levels occurred at 6 h post-exercise, significantly higher after the Yo-Yo IR2 test. All levels returned to baseline within 24 h. Findings support that intermittent, high-intensity exercise influences cardiac biomarker responses differently than continuous exercise.
Lum et al. (64)	Singapore	Compare the effects of RIST with SIST on strength, sprinting, and jump performance.	Randomized controlled trial	33 (*M* = 22; *F* = 11)	23.9 ± 3.1	Pre and post-tests: CMJ, 30 m sprint, ISqT.	6 weeks (12 sessions)	SIST led to greater improvements in peak force, rate of force development and 30 m sprint time compared to RIST. Both methods improved jump height similarly. SIST is recommended for enhancing dynamic performance in sports.
Lum et al. (65)	Singapore & USA	Compare effects of CIST and PIST on strength and dynamic performance.	Randomized controlled trial	*M* = 24	*M* = 23 ± 2.7	20 m sprint, CMJ, IMTP tests	24 weeks	Both CIST and PIST improved sprint performance and strength compared to control. CIST showed greater improvements in 5 m and 10 m sprint times, while both CIST and PIST improved 20 m sprint times. No significant differences between CIST and PIST in strength gains.
Toivo et al. (66)	Finland	Compare physical activity levels measured by accelerometery.	Cross-sectional study	332 (*M* = 159; *F* = 173)	15.6 ± 0.5	Accelerometer worn on the hip for 7 days, PA reported using 1 min exponential moving average.	7 days	MVPA was 97 ± 21 min/day for males and 83 ± 31 min/day for females. During training days, males accumulated 113 ± 33 min of MVPA. Sports participation significantly contributed to meeting PA recommendations, with 85 % of sports club participants reaching the recommended 60 min of MVPA daily vs. 45 % of non-participants.

RIST, rapid non-sustained contraction Isometric strength training; SIST, Sustained contraction Isometric strength training; CMJ, Countermovement jump; ISqT, isometric squat; CIST, consecutive 24 weeks isometric strength training; PIST, periodic inclusion isometric strength training; IMPT, isometric midthigh pull; PA, physical activity; MVPA, moderate-to-vigorous physical activity.

Two studies investigated the effects of isometric strength training on dynamic and strength performance. Lum et al. (64) reported that a six-week sustained isometric strength training (SIST) program improved peak force, rate of force development, and sprint time more than rapid isometric strength training (RIST), with both protocols improving jump height. In a 24-week intervention, Lum et al. (65) found that both continuous isometric strength training (CIST) and periodic isometric strength training (PIST) enhanced sprint performance and strength; CIST was more effective in shorter sprint distances (5 m and 10 m), while both improved 20-m sprint times.

One study (62) assessed neuromuscular coordination and strength training in a four-month quasi-experimental design. Improvements in postural stability were observed, particularly under more challenging postural and specific floorball related positions.

One study focused on asymmetry. Maly et al. (63) identified lower bilateral lower limb asymmetry in floorball players compared to controls but also found reduced knee extensor strength.

Toivo et al. (66) focused on physical activity levels, it was reported that daily moderate-to-vigorous physical activity (MVPA) levels averaged 97 ± 21 min for boys and 83 ± 31 min for girls. Sports club participants were more likely to meet PA recommendations than non-participants (85% vs. 45%).

Finally, Wedin et al. (67) examined cardiac troponin T responses to exercise. No elevation was found following continuous ergometer testing, while intermittent high-intensity exercise (Yo-Yo IR2 test) led to transient elevations in 3 out of 9 players, normalizing within 24 h.

## Skill

In the skill category (Table 4), three studies were included, originating from the Czech Republic, Switzerland, and Norway. The studies employed different methodologies, including one standardization study and two experimental studies. The number of participants ranged from 10 to 212, with a predominance of male participants across all studies.

**Table 4:** Skills studies.

Author (year)	Country	Aim/Purpose of study	Type of study	Participants		Methodology		Results
				Sample size, gender	Age	Methods	Follow-up period	
Dragounová (68)	Czech Republic	To develop and standardize a rating scale for diagnosing floorball skills in young school-age children.	Standardization study	212 (*M* = 197; *F* = 15)	6–12 years (9.28 ± 1.32)	A Guttman-type scale was created to evaluate skills in ball handling, control, and passing. The scale's reliability was validated, and inter-rater testing confirmed consistent evaluations between different raters.	–	The rating scale included 9 items with high reliability (0.81) and excellent inter-rater agreement (98.5 %). Shooting skills excluded as too difficult.
Lazzeri et al. (69)	Switzerland	Identify biomechanical predictors of wrist shot accuracy and speed, comparing two different feet positions.	Experimental study	*M* = 10	24.1 ± 3.7	12 camera motion capture system tracking kinematics during wrist shots from two feet positions.	Single session	Shot accuracy was better with feet at right angles to the target (0.22 m vs. 0.27 m). No difference in ball speed between positions. Accuracy improved with head stability on the target. Ball speed influenced by flexion of supporting leg, hip and trunk rotation, and wrist movement.
Van den Tillaar (70)	Norway	Investigate the effect of four different shooting techniques on velocity and accuracy, target height influence on shooting performance.	Experimental study	*M* = 10	*M* = 21.6 ± 3.6	Radar gun, videoanalysis.	Single session	Slap shots had the highest velocity, wrist shots the lowest. No significant differences in accuracy between techniques. Target height affected velocity, with lower targets producing faster shots.

Dragounova (68) developed and validated a nine item rating scale aimed at diagnosing floorball skills excluding shooting in school aged children (mean age 9.28 ± 1.32). The scale demonstrated high reliability (0.81) and excellent inter-rater agreement (98.5%).

Lazzeri et al. (69) examined biomechanical predictors of wrist shot performance in adult male players (mean age 24.1 ± 3.7). Shot accuracy improved when players' feet were positioned at right angles to the target. While ball speed did not vary between foot positions, it was influenced by factors such as supporting leg flexion, hip and trunk rotation, and wrist movement.

Van den Tillaar (70) investigated the velocity and accuracy of four different shooting techniques in male floorball players (mean age 21.6 ± 3.6). Slap shots produced the highest ball velocity, while wrist shots were the slowest. Accuracy did not significantly differ between shot types. Lower target height was associated with increased shot velocity.

Together, these studies highlight the importance of biomechanical factors and technique in shot performance and provide validated tools for assessing general skill proficiency in younger players.

## Equipment

One qualitative study from Sweden (Table 5) by Gabrielsson & Dolles (71) explored how equipment manufacturers and retailers contribute to the value creation and development of floorball as a new sport. The study involved 13 participants including players, coaches, club board members, manufacturers, and retailers, and used semi-structured interviews alongside market analysis. The findings showed that equipment manufacturers and retailers play a critical role in floorball's growth by co-creating value through innovation and marketing activities. Manufacturers like Unihoc and Salming introduced technological innovations (e.g., curved carbon fiber shafts, modular blade systems) and engaged in global promotion via sponsorships and training content. Retailers such as Klubbhuset used targeted digital media campaigns (e.g., instructional YouTube content with star players) to boost player engagement and product visibility. These coordinated actions fostered player development, increased merchandise sales, and enhanced the sport's international presence.

**Table 5:** Equipment study.

Author (year)	Country	Aim/Purpose of study	Type of study	Participants		Methodology		Results
				Sample size, gender	Age	Methods	Follow-up period	
Gabrielsson & Dolles (71)	Sweden	Analyse value capturing and market development	Qualitative study	13 (players, coaches, club board members, manufacturers, retailers)	-	Semi-structured interviews, market analysis.	No follow-up	Equipment manufacturers, retailers, and clubs co-create value for floorball's market growth. Cooperation is key for innovation and expansion.
								

## Nutrition

One prospective cohort study (Table 6) conducted in Poland and the Czech Republic investigated nutrition habits and body composition among 40 female floorball players during the preseason (72). The Czech players showed higher muscle mass (47.8 ± 4.2 kg) and body fat percentage (19.8 ± 5.4%) compared to Polish players (45.8 ± 4.2 kg and 18.6 ± 5.4%, respectively), despite similar BMI values. Dietary patterns also differed between the groups: Czech players consumed more whole grain products and low-fat dairy, while Polish players reported higher intake of processed meats, fried foods, and fruit juices. Additionally, Czech athletes reported more frequent strength training and greater adaptation of diet to training load. These findings underline both physiological and behavioural differences in nutrition strategies between the two national cohorts.

**Table 6:** Nutrition study.

Author (year)	Country	Aim/Purpose of study	Type of study	Participants		Methodology		Results
				Sample size, gender	Age	Methods	Follow-up period	
Białek-Dratwa et al. (72)	Poland, Czech Republic	Compare nutrition habits and body composition of female players during preseason.	Prospective cohort study	*F* = 40 (22 Polish,18 Czech players)	Poland: 22.0 ± 3.1, Czech Rep.: 21.8 ± 3.5	Dietary intake questionnaire, BIA.	No follow-up	Czech players had higher muscle mass and body fat than Polish players. The Czech players consumed a slightly higher amount of healthier products, such as whole-grain products. The Polish players took in more meat, processed products and fruit juices.

BIA, bioelectrical impedance analysis;.

## Discussion

This scoping review provides a comprehensive overview of the past decade of competitive floorball research, categorizing existing studies and highlighting key areas of focus. By synthesizing findings from multiple domains, it offers insights into the current state of knowledge in floorball and helps to uncover notable gaps. These findings serve as a foundation for informing directions for future research. A total of 55 studies published between 2014 and 2024, focusing on various aspects of competitive floorball are included in this scoping review. The included studies are classified into six thematic categories related to competitive floorball: health related aspects (*n* = 33), psychology and sociology (*n* = 11), physical conditioning and performance (*n* = 6), skills (*n* = 3), equipment (*n* = 1), and nutrition (*n* = 1).

Previously, Tervo & Nordström (1) who included nineteen floorball related studies into a systematic review, reported that most existing research focuses on sports medicine, particularly injury epidemiology. Early research on floorball injuries, particularly the study by (7) conducted in female players, identified a high incidence of both traumatic and overuse injuries. Building on this problem, more recent studies provide a broader perspective on injury patterns, consistently report that injuries to the joints and ligaments of the lower extremities are the most common in floorball. These studies include both male and female players and indicate (18, 22, 24, 25) (23, 73).

Research on preventive strategies evolves from early neuromuscular training interventions in female players (10, 11) to program named Knee Control, which demonstrate beneficial effects on injury incidence in both male and female players. However, the effectiveness of Knee Control program appears to depend on factors such as compliance and implementation in practice (31–35). In this scoping review, injury, overuse injury and injury prevention were the most common topics among included studies. This aligns with trends observed in other team sports, where injury epidemiology often constitutes a primary research interest. However, the health related category includes also the novel directions of research focusing on biomarkers (45–47), factors affecting the player illness (48, 49), and one study addressing vocal health among coaches (50).

Psychological aspects of floorball also receive increasing attention in the last decade. Earlier studies focused on social loafing. The phenomenon of social loafing suggests that individual identifiability within a team may influence perceived effort, performance, and group functioning (74). More recent research expands the topic by focusing on motivational climate, coaching and leadership, gender differences, stress, well-being, and athlete dropout (52, 53, 57, 59–61). Nevertheless, this area remains underdeveloped compared to other team sports.

In parallel, research on physiological demands evolves from isolated strength assessments such as one-repetition maximum (1RM) testing (75) to more comprehensive and sport specific approaches that better reflect the high intensity and intermittent nature of the game (64, 65, 67). Despite this progress, the available evidence remains relatively limited and topics related to the strength training, plyometric interventions (76, 77) and periodization (78) are largely missing.

Skills are covered by three studies, suggesting that floorball-specific motor learning and biomechanical analysis are still in their early stages. The included studies highlight the importance of biomechanical factors and technique in shot performance and provide validated tools for assessing general skill proficiency in younger players (68–70). There are no studies analysing small sided games parts of trainings, despite their established role in technical and tactical training across team sports (79) including floorball. The categories equipment (71) and nutrition (72) are each represented by one study, further emphasizing the thematic imbalance in floorball research.

Since 2014, research on floorball has expanded across multiple domains, including injury epidemiology, injury prevention, overuse injuries, physiological and performance characteristics, as well as psychological and social aspects of the sport. More recently, studies have begun to address a wider range of topics such as pain, assessment of physical functions, skill specific performance and shooting techniques, nutrition, and coaching related factors. Despite this increasing diversity, the overall body of literature remains limited, with many topics represented by only a small number of studies. Compared to other team sports of similar popularity, such as basketball, ice hockey, football, or volleyball, floorball research remains underdeveloped both in scope and volume. Therefore, there was a need for a comprehensive synthesis of existing evidence to better understand current trends and identify areas for future research.

Strengths of this scoping review include providing a comprehensive overview of the number and types of studies focused on competitive floorball in past decade, along with a synthesis of the most relevant findings. This approach enables the mapping of research areas which have already been addressed and to highlight those that remain underexplored particularly in comparison with other sports. Substantial gaps are identified both in the volume and variety of existing literature. Especially, many aspects critical to athlete development such as recovery strategies, tactical analysis, psychology of performance, or female athlete specific training needs remain underrepresented.

A limitation of this scoping review is the exclusion of studies that combined data from multiple sports without presenting separate findings for floorball, which was particularly common in cross-sectional designs. Furthermore, studies that do not specify the competitive level of the participants are excluded. Only articles published in English and Czech were included, and relevant research published in other languages or in the grey literature may have been missed. Finally, as per the scoping review methodology, no formal quality assessment of included studies is conducted.

## Conclusion

This scoping review provides a comprehensive overview of research on competitive floorball over the last decade, highlighting the main areas of focus and identifying key gaps in the literature. The findings show that current research is predominantly centered on injury epidemiology and prevention, while other important domains such as physiological demands, performance, psychological aspects, and skill development remain relatively underexplored. Compared to other team sports of similar popularity, the overall volume and diversity of floorball research are still limited. This highlights the need for further studies, particularly those adopting longitudinal, multidisciplinary, and sport-specific approaches. Future research should focus on expanding underrepresented areas, improving methodological quality, and addressing practical applications relevant to coaches and athletes. Overall, the present review contributes to a better understanding of the current state of floorball research and provides a foundation for the continued development of this sport within the field of sport science.

## Data Availability

The original contributions presented in the study are included in the article/Supplementary Material, further inquiries can be directed to the corresponding author/s.
